# Exploration of fever characteristics in parturients under continuous temperature monitoring during labor analgesia and analysis of the impact on maternal and neonatal outcomes: an observational study

**DOI:** 10.3389/fgwh.2025.1541227

**Published:** 2025-04-29

**Authors:** Xia Li, Junli Ma

**Affiliations:** ^1^ Department of Anesthesiology, Sichuan Academy of Medical Sciences & Sichuan Provincial People's Hospital, Chengdu, China; ^2^ Department of Anesthesiology, West China Hospital of Sichuan University, Chengdu, China

**Keywords:** continuous temperature monitoring, labor analgesia, fever, characteristics, outcomes

## Abstract

**Objective:**

By continuous core temperature monitoring, this study aims to explore the patterns of fever in parturients receiving labor analgesia and analyze its impact on both the maternal and neonatal.

**Methods:**

Non-invasive temperature monitoring sensors (iThermonitor705) and labor analgesia temperature management system were used to collect temperature data from parturients. Based on the presence or absence of fever during the peripartum period, the subjects were divided into a fever group and a non-fever group. Maternal temperature data during the peripartum period, along with maternal and neonatal demographic and clinical characteristics, were collected.

**Results:**

Among the parturients receiving labor analgesia, 25.9% (43/166) developed a fever during the peripartum period. Of these, 25.6% (11/43) experienced their first fever after delivery, all occurring within 2 h after delivery. Compared to the non-fever group, the fever group had longer durations of the first stage of labor, total labor duration, and epidural analgesia. Additionally, the fever group had a higher rate of meconium-stained amniotic fluid (grade III), used more analgesics, and had a higher rate of antibiotic use. However, no significant differences in adverse maternal and neonatal outcomes were observed between the two groups.

**Conclusion:**

Fever can occur not only during labor but also for the first time after delivery. Although fever during the peripartum period increases the rate of maternal exposure to antibiotics, there were no significant differences in maternal or neonatal outcomes between the two groups.

## 1 Introduction

To reduce the pain of parturients during labor, epidural analgesia has become the preferred option to optimize the birthing experience (1). However, it is associated with a fever rate of about 20%–40% (2–4), commonly referred to as intrapartum fever. The potential adverse effects of intrapartum fever on both the mother and the baby remain controversial. Some studies suggest that it may increase the rates (2, 5–7) of emergency cesarean section, dystocia, and postpartum hemorrhage in mothers, while in newborns (6, 7), it may lead to low Apgar scores, respiratory distress, hypotonia, neonatal brain injury, and even cerebral palsy, which may lead to its low Apgar score, respiratory distress, low tension, neonatal brain injury, and even cerebral palsy. However, other research (4) indicates that maternal fever has no significant adverse impact on maternal or neonatal outcomes.

Maternal fever can be caused by both infectious and non-infectious factors, with most cases of fever following epidural analgesia being attributed to non-infectious causes clinically (3). The exact mechanism of fever is still unclear but may be related to dysregulation of the thermoregulatory center, labor analgesia, elevated environmental temperature, maternal dehydration, or activation of the pro-inflammatory cascade (2, 8). Studies (2, 3, 7) have also found that parturients with premature rupture of membranes, longer durations of epidural catheterization or labor, increased use of analgesics, and more frequent vaginal examinations are more prone to developing fever. Current methods of monitoring maternal temperature rely mainly on fixed intervals (4) or cervical dilation progress (9), which makes it difficult to obtain dynamic temperature information and promptly detect fever in parturients. Therefore, optimizing methods for temperature monitoring and exploring the patterns of maternal fever can help provide special attention to mothers who are experiencing or at high risk of developing a fever, thus minimizing the occurrence of adverse maternal and infant events.

This study introduces the use of a wireless, non-invasive temperature monitoring sensor (iThermonitor705) based on the Internet, which has been proven to accurately reflect patients' core temperature (10, 11). Combined with a labor analgesia temperature management system, this allows for continuous, real-time temperature monitoring in parturients, enabling earlier detection of abnormal temperatures and more precise identification of fever patterns and characteristics. Additionally, this study aims to explore the impact of peripartum fever on maternal and neonatal complications, providing clues for standardized clinical management of febrile parturients and laying the groundwork for investigating the mechanisms of peripartum fever.

## 2 Methods

### 2.1 Study design and setting

This dual-center case-control study was conducted in Sichuan Provincial People's Hospital and Jinjiang Maternity and Child Health Care Hospital from January 2021 to August 2021. The research protocol was approved by the Ethics Committee of Sichuan Provincial People's Hospital [Protocol No.: 2020356] and the Ethics Committee of Jinjiang Maternity and Child Health Care Hospital [Protocol No.: 2021010], and registered in the Chinese Clinical Trial Registry (Registration No.: ChiCTR2000037802).

### 2.2 Participants and sampling

This study enrolled full-term parturients who underwent painless delivery at Sichuan Provincial People's Hospital and Jinjiang Maternity and Child Health Care Hospital. Inclusion criteria were as follows: age ≥18 years; no contraindications to epidural puncture; admission to the delivery room with cervical dilation ≤3 cm. Exclusion criteria were as follows: an axillary temperature ≥38 ℃ prior to inclusion in the study; recent use of steroid hormones; use of antipyretics within the past week; factors affecting the accuracy of axillary temperature monitoring, such as adhesive allergies, absence of both upper limbs, and hyperhidrosis; contraindications to epidural puncture. Withdraw criteria were as follows: conversion to cesarean section during labor; voluntary withdrawal from the study; interruption of axillary temperature monitoring for ≥30 min during labor. Parturients were allocated into fever and non-fever groups based on the presence of fever (axillary temperature ≥38 ℃) during the peripartum period.

### 2.3 Study procedure and analgesia protocol

**Figure 1:**
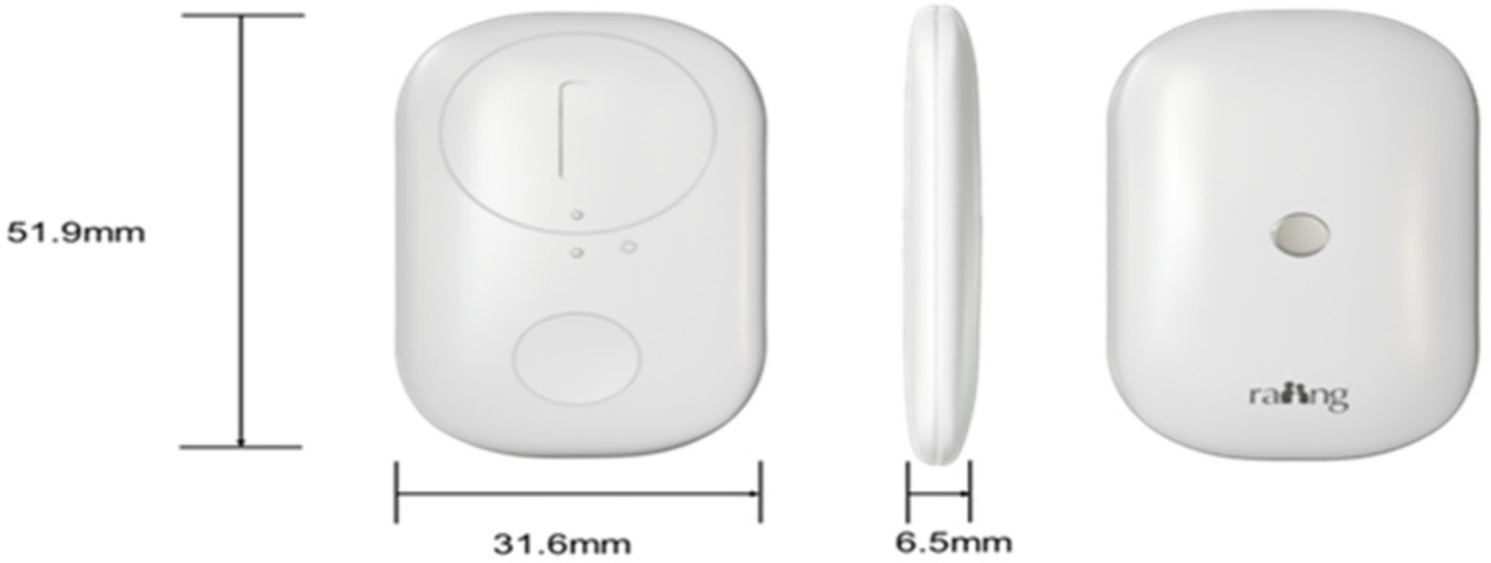
Temperature monitoring sensor (iThermonitor 705); 52 mm × 32 mm × 6.5 mm, weight approximately 7 g (including batteries).

After parturients meeting the inclusion criteria are admitted to the delivery room and have signed the informed consent form, the axillary hair is immediately shaved. A temperature monitoring sensor (iThermonitor 705; Raiing Medical, China, Figure 1) is then attached to the lateral chest wall of the axilla. The parturients are instructed to keep the axilla on that side tightly closed for 5–10 min. Once the central control computer of the labor analgesia temperature management system displays stable and high-quality temperature data (stable = 2), the parturients are allowed to move the arm on that side freely. This device transmits the obtained temperature data (one data point every 4 s) via Bluetooth to a signal repeater, which then transmits the average temperature for each minute to the central control computer of the labor analgesia temperature management system. In this way, we can obtain real-time temperature data of the parturients.

Continuous temperature monitoring for parturients starts from the time they are admitted to the delivery room until 2–12 h after delivery. Decisions regarding the administration of labor analgesia and the management of maternal fever are made by the attending obstetrician, while the researchers are only responsible for collecting relevant data.

Upon receiving the obstetrician's order for labor analgesia, an intravenous line is immediately opened (avoiding the same side as the temperature monitoring and infusion). Emergency medications are prepared bedside, and the parturient is connected to an electrocardiogram monitor and given oxygen via nasal cannula. The parturient is then assisted into a left lateral knee-chest position, and an epidural puncture is performed at the L3–4 level. The anesthesiologist selects one of the following anesthesia regimens: Regimen 1 (epidural labor analgesia): After a successful epidural puncture, an epidural catheter is inserted 3–5 cm towards the head. The parturient is then placed in the supine position, and after confirming no abnormalities upon catheter aspiration, a test dose of the drug is administered. After 5 min of observation to confirm the catheter's position, the Programmed Intermittent Epidural Bolus (PIEB) is connected and the first dose is administered; Regimen 2 (combined spinal-epidural analgesia): After a successful epidural puncture, a subarachnoid puncture is performed, and 2 ml of a solution containing 0.1% Ropivacaine is administered. The epidural catheter is then inserted 3–5 cm towards the head. Similarly, after placing the parturient in the supine position and confirming no abnormalities upon catheter aspiration, a test dose of the drug is administered. After 5 min of observation to confirm the catheter's position, the PIEB pump is connected, and 30 min after the subarachnoid drug administration, the PIEB pump is activated. The analgesic formula consists of 222 ml of normal saline + 200 mg ropivacaine + 100 ug sufentanil. The pulse dose is 10 ml/h, with a lockout time of 20 min, and an additional dose of 5 ml. The test dose is 3 ml of 1.5% Lidocaine.

If the parturient experiences paresthesia during the puncture or catheter insertion, the needle will be immediately withdrawn, and the puncture will be attempted again. If two punctures fail, another anesthesiologist will perform the procedure. If three punctures fail, the parturient will be advised to choose an alternative analgesia method, and she will be excluded from the study.

This study is an observational study, and all data are collected after delivery through the electronic medical record system or by telephone follow-up three months after delivery. Temperature data will be exported uniformly after the study and verified again for completeness.

### 2.4 Data collection and statistical analysis

The following data were collected, including the primary study aim (the rate of peripartum fever), secondary study aims (the temperature information of the parturients, delivery-related information, and adverse complications of parturients and infants), maternal demographic and clinical characteristics [age, height, weight, BMI, gestational age, pregnancy complications, duration of labor, duration of epidural analgesia, amount of analgesics used, rate of meconium-stained amniotic fluid (grade III), and postpartum adverse events], and neonatal demographic and clinical characteristics (Apgar scores, NICU admission rate after delivery, NICU length of stay, and neonatal pneumonia incidence). For short-term adverse events for infants and mothers, telephone follow-up was conducted three months after delivery.

Based on previous literature (12) and the maternal fever rate in our hospital, the probability of intrapartum fever in parturients was estimated to be approximately 25%. Based on previous finding and the following formula $nC=\frac{(r+1){\left(z1-\beta +z1-\alpha \right)}^{2}S^{2}}{r{\left(\Delta -\delta \right)}^{2}}$ with *α* = 0.05 and power = 0.90, we obtained a calculated minimum sample size of 111 parturients for the non-fever group and 37 parturients for the fever group. Considering that in the pre-experiment, 25% of parturients receiving labor analgesia may switch to cesarean section for various reasons, and 15% may withdraw from the study due to incomplete temperature monitoring caused by sensor detachment, a final total of 208 cases were included in the study.

Statistical analysis was performed using SPSS 25.0 software. The Shapiro–Wilk test was used to determine the normality of continuous variables. (1) For categorical variables, percentages (%) were used for statistical description, and Chi-square or Fisher's exact tests were used for statistical analysis; (2) For continuous variables, depending on the normality of the data, mean ± standard deviation or median (interquartile range) was used for statistical description, and *t*-tests, analysis of variance (ANOVA), and non-parametric tests were used for statistical analysis. *P*-value <0.05 was considered statistically significant.

## 3 Results

**Figure 2:**
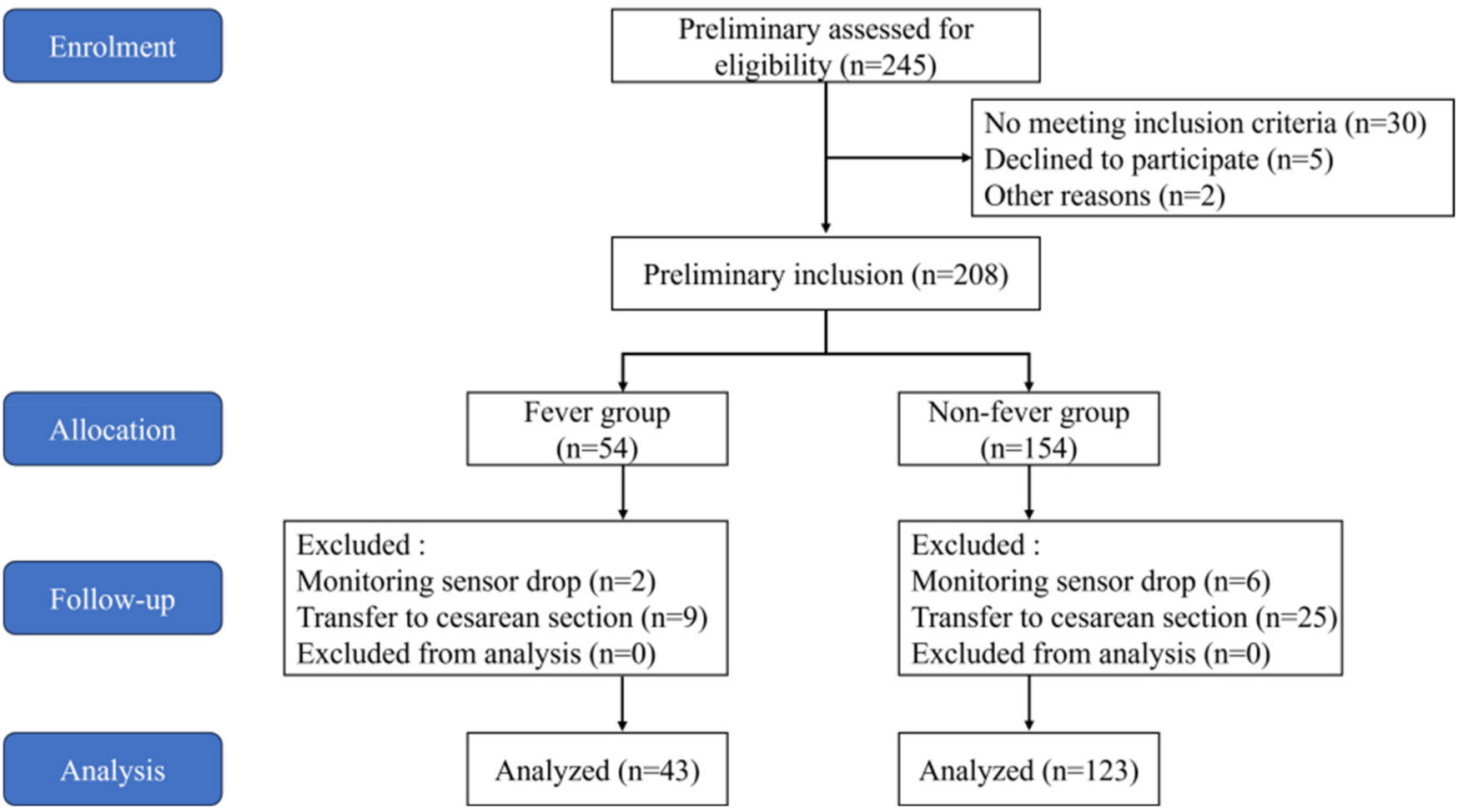
Flowchart of inclusion and exclusion it includes the entire study from patient enrolment, screening, allocation, follow-up, and final inclusion analysis.

A total of 245 parturients were screened, of which 208 met the inclusion criteria for the study, and 166 completed the study (Figure 2). Based on the presence of fever, the parturients were divided into a fever group and a non-fever group, with no significant difference in dropout rates between the two groups. Among them, 43 parturients (25.9%) developed a fever during the peripartum period, while 123 parturients (74.1%) did not. The two groups were balanced in terms of sociodemographic characteristics, complications, and delivery data, with no statistically significant differences observed (*P* > 0.05) (Supplementary Table S1).

### 3.1 Fever characteristics

Out of the 166 parturients included in the study, 43 developed fever during the peripartum period (axillary temperature ≥38 °C), with an incidence rate of 25.9% (43/166). The fever rate among parturients receiving epidural labor analgesia was 20% (12/60), while it was 29.2% (31/106) among those receiving combined spinal-epidural analgesia. Fever could occur at any point during labor; some parturients even developed fever after delivery, accounting for 25.6% (11/43) of all febrile cases, which we defined as postpartum fever. Among parturients developing fever, 72.7% (8/11) developed fever within 1 h postpartum, with the latest onset of fever at 1.7 h postpartum. The mean duration of postpartum fever was 2.1 h. For women experiencing intrapartum fever, the mean duration of fever was 2.7 h. Additionally, 71.9% (23/32) of parturients with fever still had fever after delivery, where delivery completion was defined as placental expulsion. Among these parturients, the mean time from delivery completion to fever resolution was 1.6 h (*n* = 23). In the fever group, the average duration from epidural catheter placement to the onset of fever was 7.0 h.

### 3.2 Clinical characteristics

#### 3.2.1 Labor events

Compared to non-fever parturients, febrile parturients had a longer first stage of labor [600.0 (420.0, 825.0) min vs. 500.0 (320.0, 750.0) min; *P* = 0.034], longer total labor duration [660. 0 (490.0, 877.0) min vs. 560.0 (387.0, 832.0) min; *P* = 0.039], and longer duration of epidural analgesia [8.32 (6.60, 11.20) h vs. 6.25 (4.5, 10.50) h; *P* = 0.008]. They also had a higher rate of meconium-stained amniotic fluid (grade III) [8 (18.6%) vs. 8 (6.5%); *P* = 0.033] and used more analgesics [79 (58, 92) ml vs. 56 (37, 92) ml; *P* = 0.005]. However, there were no significant differences between the two groups in terms of the duration of the second stage of labor or premature rupture of membranes (*P* > 0.05) (Table 1).

**Table 1:** Comparison of pregnancy-related conditions during labor between the Two groups of parturients.

Factors	Fever group	Non-fever group	*P*
Duration of the first stage of labor (min)	600.0 (420.0, 825.0)	500 (320.0, 750.0)	0.034^a^
Duration of the second stage of labor (min)	54.0 (35.0, 80.0)	46.0 (25.0, 86.0)	0.162
Duration of the third stage of labor (min)	5.0 (5.0, 7.0)	6.0 (5.0,8.0)	0.108
Total labor duration (min)	660.0 (490.0, 877.0)	560.0 (387.0, 832.0)	0.039^a^
Grade III meconium-stained amniotic fluid *n* (%)	8 (18.6%)	8 (6.5%)	0.033^a^
Premature rupture of membranes *n* (%)	16 (37.2%)	41 (33.3%)	0.645
Duration of epidural analgesia (h)	8.3 (6.6, 11.2)	6.3 (4.5, 10.5)	0.008^a^
Analgesic dosage (ml)	79 (58, 92)	56 (37, 92)	0.005^a^
Duration from membrane rupture to fetal delivery (h)	10.65 ± 11.83	9.94 ± 13.94	0.218

^a^ Compared with the no-fever group, *P* < 0.05.

#### 3.2.2 Maternal and neonatal adverse events

In terms of maternal outcomes, aside from the significantly higher antibiotic usage rate in the fever group (65.7% vs. 47.2%, *P* = 0.042), there were no statistically significant differences in maternal outcome indicators between the two groups (Table 2).

**Table 2:** Comparison of postpartum complications in the two groups of parturients.

Factors	Fever group (43)	Non-fever group (123)	*P*
Immediate postpartum hemorrhage (ml)	200 (200,300)	200 (200,270)	0.379
Hemorrhage at 2 h postpartum (ml)	85 (60,100)	70 (50,100)	0.081
Postpartum hemorrhage *n* (%)	5 (11.6%)	7 (5.7%)	0.301
Postpartum curettage *n* (%)	0 (0%)	7 (5.7%)	0.192
Postpartum uterine atony *n* (%)	6 (14.0%)	8 (6.5%)	0.198
Post delivery antibiotic use *n* (%)	28 (5.1%)	58 (47.2%)	0.042*
Hospitalization days after delivery (day)	1.77 (1.56,2.3)	1.82 (1.49,2.29)	0.204
Re-hospitalization 3 months after delivery *n* (%)	1 (2.3%)	3 (2.4%)	>0.999

* Compared with the no-fever group, *P* < 0.05.

Regarding neonatal outcomes, there were no statistically significant differences between the two groups in Apgar scores after birth, NICU admission rates, NICU length of stay, incidence of neonatal pneumonia, or neonatal readmission rates within three months postpartum (Supplementary Table S2).

## 4 Discussion

In our study, we used a non-invasive continuous core temperature monitoring sensor (iThermonitor705) to collect temperature data from parturients and investigate the patterns of fever and its impact on maternal and neonatal outcomes. Similar to other studies (2, 4) the incidence of fever in our parturients was 25.9%. Additionally, we found that fever might occur at delivery in parturients receiving labor analgesia and for some, might occur for the first time in the postpartum period. Most fevers occurring after the onset of the deceleration phase. Regarding maternal and neonatal outcomes, apart from an increased rate of antibiotic exposure in febrile parturients, we did not observe a significant impact of fever on the mother and newborn.

Like most studies (2, 5, 9), we defined maternal fever as a peripartum body temperature ≥38 °C. The mechanism of peripartum fever in parturients remains unclear. Infectious causes of fever include chorioamnionitis, urinary tract infection, and respiratory tract infection (8), while potential non-infectious causes (2, 7, 8) may include dysregulation of the thermoregulatory center, labor analgesia, increased environmental temperature, maternal dehydration, and activation of the pro-inflammatory cascade. Among these, the activation of the pro-inflammatory cascade triggering maternal fever is considered the most likely mechanism: levels of IL-6, white blood cells, neutrophils, the neutrophil-to-lymphocyte ratio, and other markers (3, 13, 14) are higher in febrile parturients prior to the onset of labor. The duration of labor may be prolonged following epidural analgesia (2, 3, 15), and as labor progresses, factors such as increased epidural catheter duration, higher doses of narcotic analgesics, and increased frequency of vaginal examinations may elevate pro-inflammatory factors, supporting cytokine activation and enhancing the response to aseptic inflammatory processes (16), thus leading to maternal fever. Some studies suggest that the use of local anesthetics for labor analgesia may activate non-infectious inflammatory responses through mechanisms such as inducing oxidative stress and cellular damage (17), resulting in maternal fever. The inability of prophylactic antibiotic use (18) and acetaminophen (19) to reduce maternal fever rates also indirectly suggests that fever in parturients may be non-infectious in origin. In summary, the mechanism of peripartum fever in parturients receiving routine labor analgesia remains unclear and requires further exploration. However, identifying fever characteristics and intervening at key points may help improve maternal and neonatal outcomes related to fever.

Current temperature monitoring methods for parturients are mostly based on manual measurements taken at fixed time intervals (4, 9), with continuous, uninterrupted measurement techniques being rare. Reducing the interval between measurements increases clinical workload. This study is the first in China to introduce a non-invasive temperature monitoring sensor (10, 11) and a labor analgesia temperature management system. This device utilizes the latest artificial intelligence algorithms to optimize for potential interferences across various application scenarios, achieving a high level of temperature monitoring accuracy and has been confirmed to reflect core temperature. The device records temperature data every 4 s and uploads the averaged minute-by-minute temperature data to the labor analgesia temperature management system, enabling us to obtain immediate and continuous core temperature data. Compared with conventional temperature monitoring methods, this technology allows for earlier detection of abnormal temperature patterns in parturients and a more comprehensive exploration of peripartum temperature characteristics in labor analgesia.

Similar to other studies (2, 4), this study observed an overall fever rate of 25.9% among parturients, with a fever rate of 20% in those undergoing epidural analgesia and 29.2% in those receiving combined spinal-epidural analgesia. Some studies (9, 20) using intermittent temperature monitoring found a trend of gradual temperature increase as labor progressed. However, few studies have used continuous monitoring devices to track core temperatures continuously through the postpartum period. Fever may occur at any stage of labor, but this study uniquely found that parturients may also experience a first fever after labor, defined here as postpartum fever, accounting for 25.6% (11/43) of all fever cases. All postpartum fevers began within around 1–2 h after delivery, with temperatures in all parturients not yet normalized at the end of labor returning to normal within an average of 2 h postpartum. However, no high-risk factors or adverse maternal or neonatal outcomes associated with postpartum fever were observed in this study, which may be attributed to the limited sample size, underlining the need for larger-scale studies to further explore the characteristics and potential maternal and neonatal complications of postpartum fever. Therefore, extending maternal temperature monitoring beyond the end of labor may aid in timely fever detection and management.

Among parturients receiving labor analgesia, those with fever had a longer first stage of labor, longer total labor time and epidural catheterization time, and a higher incidence of amniotic fluid staining (grade III) compared to those with no fever, similar prior research findings (2, 20). This phenomenon may be associated with the activation of inflammatory factors and the cascade of pro-inflammatory reactions in the body (2, 3, 15, 16). There is no consensus on the impact of epidural-related fever during labor on maternal and neonatal outcomes. Fever (2, 5, 7) may increase cesarean section rates, the incidence of labor dystocia, instrumental deliveries, and antibiotic use for mothers; for neonates, it (5, 6) may contribute to low Apgar scores, respiratory distress, hypotonia, neonatal brain injury, and even cerebral palsy. However, some studies (4) indicate no significant differences in maternal and neonatal outcomes associated with peripartum fever. Our study found that aside from an increased maternal antibiotic exposure rate, there were no significant differences in outcome measures between the fever and non-fever groups. This may be because our study promptly detected and managed fever, reducing adverse events. However, as this is an observational study without interventions in clinical decision-making by obstetricians, certain confounding factors may have affected the results.

This study has both strengths and limitations. Its strengths lie in the pioneering use of wireless, non-invasive temperature monitoring sensors (iThermonitor705) (10, 11) and a labor analgesia temperature management system to collect continuous, real-time core temperature curves from parturients and to extend temperature monitoring to 2–12 h postpartum. This approach enables dynamic monitoring and a deeper exploration of maternal temperature patterns, offering insights into the adverse impact of peripartum fever on maternal and neonatal outcomes and providing a foundation for studying the mechanisms of peripartum fever. However, the study also has limitations. First, as an observational study, it lacks standardized fever management protocols for parturients, and varying treatment approaches inevitably introduced confounding factors. Additionally, no standardized anesthesia and analgesia protocols were established in the present study, which caused potential biases in the analysis of results. Second, the study did not incorporate laboratory tests, case history, or PCR results, limiting the exploration of fever etiology at a mechanistic level. Third, due to technical constraints and equipment limitations, the postpartum temperature monitoring time was neither standardized nor consistently extended for all participants, which may have led to the undetection of some cases of postpartum fever, thereby limiting the comprehensive assessment of postpartum fever patterns. In future studies, the follow-up of mothers and neonates should be extended to assess the long-term effects of fever on maternal and neonatal complications.

## 5 Conclusion

This study innovatively found that parturients may not only experience fever during labor but may also develop a first fever after labor, with all postpartum fevers occurring within 2 h after delivery. Although fever increases maternal antibiotic exposure, no significant adverse effects on maternal or neonatal outcomes were observed.

## Data Availability

The raw data supporting the conclusions of this article will be made available by the authors, without undue reservation.
