# Supplementary material for: Exploration of fever characteristics in parturients under continuous temperature monitoring during labor analgesia and analysis of the impact on maternal and neonatal outcomes: an observational study
Source: Front Glob Womens Health. 2025 Apr 29;6:1541227. doi: 10.3389/fgwh.2025.1541227 (PMC12069292; doi:10.3389/fgwh.2025.1541227)
Supplement: Supplementary file 1 [file Datasheet1.docx]

Supplementary Material

# Supplementary Data

All research data can contact the author to obtain

# Supplementary Figures and Tables

## Figures


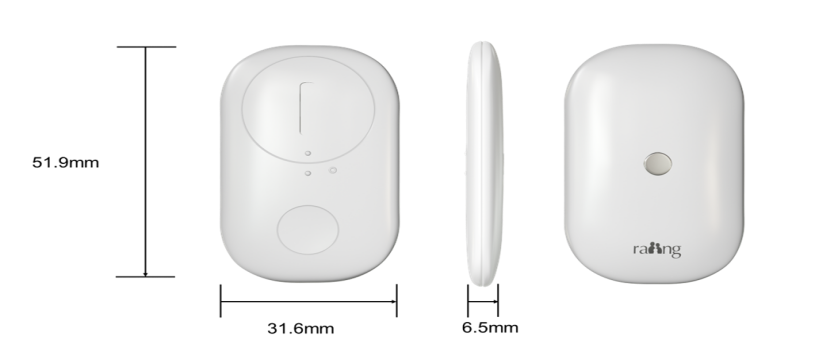


figure 1: The Temperature Monitoring Sensor (iThermonitor 705), 52*32*6.5mm, weight approximatelt 7g (including batteries)


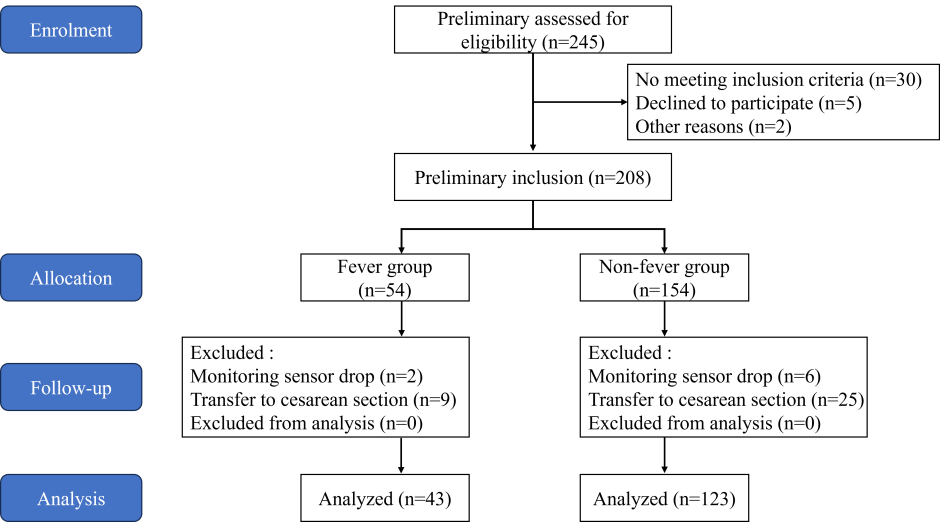


Figure 2: Flowchart of Inclusion and Exclusion

It includes the entire study from patient enrolment, screening, gallocation, follow-up, and final inclusion analysis.

## Tables

## Table 1. Comparison of Pregnancy-Related Conditions During Labor between the Two Groups of Parturients

|  | Fever Group(43) | Non-Fever Group(123) | *P* |
| --- | --- | --- | --- |
| Duration of the first stage of labor (min) | 600.0 (420.0, 825.0) | 500 (320.0, 750.0) | 0.034**^a^** |
| Duration of the second stage of labor (min) | 54.0 (35.0, 80.0) | 46.0 (25.0, 86.0) | 0.162 |
| Duration of the third stage of labor (min) | 5.0 (5.0, 7.0) | 6.0 (5.0,8.0) | 0.108 |
| Total labor duration (min) | 660.0 (490.0, 877.0) | 560.0 (387.0, 832.0) | 0.039**^a^** |
| Grade III meconium-stained amniotic fluid n (%) | 8 (18.6%) | 8 (6.5%) | 0.033**^a^** |
| Premature rupture of membranes n (%) | 16 (37.2%) | 41 (33.3%) | 0.645 |
| Duration of epidural analgesia (h) | 8.3 (6.6, 11.2) | 6.3 (4.5, 10.5) | 0.008**^a^** |
| Analgesic dosage (ml) | 79 (58, 92) | 56 (37, 92) | 0.005**^a^** |
| Duration from membrane rupture to fetal delivery (h) | 10.65 ± 11.83 | 9.94 ± 13.94 | 0.218 |

note: Compared with the no-fever group, ^a^P < 0.05

**Table2: Comparison of postpartum complications between the two groups**

|  | Fever Group(43) | Non-Fever Group(123) | P |
| --- | --- | --- | --- |
| Immediate postpartum hemorrhage（ml） | 200（200,300） | 200（200,270） | 0.379 |
| Haemorrhage at 2h postpartum（ml） | 85（60,100） | 70（50,100） | 0.081 |
| postpartum hemorrhage n(%) | 5（11.6%) | 7（5.7%) | 0.301 |
| Postpartum curettage(%) | 0（0%) | 7（5.7%) | 0.192 |
| Postpartum uterine atony n(%) | 6（14.0%) | 8（6.5%) | 0.198 |
| Post delivery antibiotic use n(%) | 28（5.1%） | 58（47.2%） | 0.042* |
| Hospitalization days after delivery（day） | 1.77（1.56,2.3） | 1.82（1.49,2.29） | 0.204 |
| Re-hospitalization 3 months after delivery n(%) | 1（2.3%) | 3（2.4%) | ＞0.999 |

note: Compared with the no-fever group, *P < 0.05.

## Supplementary Figures

## Supplementary Tables

Table S1：sociodemographic characteristics between the two groups of parturients

|  | Fever Group（43） | Non-Fever Group(123） | P |
| --- | --- | --- | --- |
| Age(years) | 27.86±3.55 | 28.72±3.58 | 0.175 |
| BMI(kg/m2) | 25.61±3.24 | 25.94±2.91 | 0.534 |
| Neonatal birth weight(g) | 3167.67±416.02 | 3173.94±358.89 | 0.925 |
| Gestational age(weeks) | 39（38,40） | 39（39,40） | 0.381 |
| Parturients complications n(%) |  |  |  |
| Gestational diabetes mellitus n(%) | 10（23.3%） | 29（23.6%） | 0.966 |
| Gestational hypertention n(%) | 2（4.7%） | 4（3.3%) | 0.650 |

note: Compared with the non-fever group, ^a^P < 0.05.

Table S2: neonatal outcomes between the two groups

|  | Fever Group (43) | Non-Fever Group(123) | P |
| --- | --- | --- | --- |
| 1min Apgar scores | 9.24±0.492 | 9.32±0.693 | 0.594 |
| 5min Apgar scores | 9.98±0.152 | 9.97±0.178 | 0.751 |
| The rate of Admission to neonatal ward n(%) | 10（23.3%） | 27（22.0%) | 0.860 |
| The days of Admission to neonatal ward* | 3（2，5） | 5（3,7） | 0.068 |
| Neonatal pneumonia n (%) | 1（2.3%) | 7（5.7%) | 0.682 |
| Readmission rate 3 months after delivery n(%) | 0（0%) | 1（0.8%) | 0.921 |

note: Compared with the no-fever group, ^a^P < 0.05.
